# Tuberculosis after Preventive Therapy in Persons Living with HIV Initiating Antiretroviral Therapy

**DOI:** 10.3201/eid3208.260493

**Published:** 2026-08

**Authors:** Qiao Chen

**Affiliations:** The Fourth Affiliated Hospital of Southwest Medical University, Sichuan, China

**Keywords:** Tuberculosis and other mycobacteria, HIV/AIDS and other retroviruses, bacteria, viruses, tuberculosis preventive therapy, antiretroviral therapy, Mozambique

**To the Editor:** I read with interest the article by Templin et al. examining tuberculosis (TB) after TB preventive therapy (TPT) among persons living with HIV recently initiating antiretroviral therapy (ART) in Mozambique (*1*). The question is intriguing, but several design and reporting choices complicate causal interpretation.

First, exposure was defined by eventual TPT completion (>170 days of isoniazid or >80 days of 3HP [12-dose isoniazid/rifapentine regimen]), whereas follow-up began at ART initiation. Classifying persons at time zero by information known only after surviving long enough to complete treatment can introduce immortal time bias unless completion is modeled as time-varying (*2*). The longer median time to TB diagnosis among those who complete therapy might reflect analytic structure as well as biology. That bias might be limited if TB during TPT is uncommon, but its magnitude cannot be inferred from the tables. Treating TPT status as time-varying, or performing landmark or sensitivity analyses excluding early follow-up, would test robustness.

Second, routine programmatic data remain useful despite missing data and imperfect timing. HIV viral load and CD4 results describe clinical status, but their timing relative to TB diagnosis and missing CD4 data should be considered cautiously in causal models. Clarifying the discrepant ART-pickup exclusion windows (3 months in Methods versus 30 days in Results) would improve reproducibility (*1*).

Those issues do not challenge the established benefit of TPT given with ART (*3*). Rather, they suggest uncertainty about the magnitude and timing of benefit in this analysis. Because randomized evidence did not show added benefit from annual repeat 3HP in Ethiopia, Mozambique, and South Africa (*4*), conclusions about a second TPT course after 1 year should remain cautious pending analyses less susceptible to immortal time bias.
